# Liver Metabolic Alterations and Changes in Host Intercompartmental Metabolic Correlation during Progression of Malaria

**DOI:** 10.1155/2011/901854

**Published:** 2011-05-29

**Authors:** Arjun Sengupta, Angika Basant, Soumita Ghosh, Shobhona Sharma, Haripalsingh M. Sonawat

**Affiliations:** ^1^Department of Chemical Sciences, Tata Institute of Fundamental Research, Homi Bhabha Road, Colaba, Mumbai, Maharashtra 400005, India; ^2^Department of Biological Sciences, Tata Institute of Fundamental Research, Homi Bhabha Road, Colaba, Mumbai, Maharashtra 400005, India; ^3^The Division of Biological Sciences, University of Chicago, 5812 S. Ellis Avenue, Chicago, IL 60637, USA

## Abstract

^1^H NMR-based metabonomics was used to investigate the multimodal response of mice to malarial parasite infection by *P. berghei* ANKA. Liver metabolism was followed by NMR spectroscopy through the course of the disease in both male and female mice. Our results showed alterations in the level of several metabolites as a result of the infection. Metabolites like kynurenic acid, alanine, carnitine, and *β*-alanine showed significant alteration in the liver, suggesting altered kynurenic acid, glucose, fatty acid and amino acid pathways. Distinct sexual dimorphism was also observed in the global analysis of the liver metabolic profiles. Multiway principal component analysis (MPCA) was carried out on the liver, brain, and serum metabolic profile in order to explore the correlation of liver and brain metabolic profile to the metabolite profile of serum. Changes in such correlation profile also indicated distinct sexual dimorphism at the early stage of the disease. Indications are that the females are able to regulate their metabolism in the liver in such a way to maintain homeostasis in the blood. In males, however, choline in liver showed anticorrelation to choline content of serum indicating a higher phospholipid degradation process. The brain-serum correlation profile showed an altered energy metabolism in both the sexes. The differential organellar responses during disease progression have implications in malaria management.

## 1. Introduction

*Plasmodium *is the organism responsible for malaria. It affects 200–300 million people and leads to a million deaths annually [1], thereby posing a global threat. The clinical symptoms of the infection are manifested during the blood stage of the parasite life cycle in the human host [2]. In acute stages of the disease, more than one tissue types are known to be affected [2, 3]. It was shown that during this stage of the disease, the parasitized RBCs show sequestration thereby affecting the microvasculature of heart, kidney, liver, intestines, adipose tissues, and eyes [4–6]. This may result in localized metabolic stress. As the disease progresses towards severity, several complications arise, possibly due to the inflammatory immune response of the host which result in complications such as liver damage, renal failure, cerebral malaria, hypoglycemia and acidosis, some of which may lead to death [7–9]. Although, the transition into these conditions is poorly understood, all of them are associated with different metabolic complications. Thus, one approach towards understanding the disease progression is to understand the metabolic alterations that occur as the disease progresses towards severe stage. These alterations can be monitored at single tissue or biofluid level and/or at the level of the intertissue and/or tissue-biofluid correlation, where one focuses on more than one tissue/biofluid simultaneously. 

^1^H NMR spectroscopy of tissues and biofluids followed by multivariate statistical analyses in a systems biological approach has been widely used in understanding metabolic changes [10]. ^1^H NMR spectra provide an unbiased profile of metabolites present in the tissue or biofluids thereby providing a good platform for the study of different conditions like breast cancer, diabetes, coronary artery disease, and high blood pressure [11–14]. Metabolic response towards parasitic infections has also been reported in the mouse models of *Trypanosome *and *Schistosome *infection [15, 16]. Recently, our group has shown relevant metabolic links and distinct sexual dimorphism with the progression of malaria in the mouse model by NMR spectroscopy and multivariate statistical analysis on urine, sera, and brain [17].

^1^H NMR spectra from a biofluid like serum or urine at a single time point provide the snapshot of the molecular phenotype or metabotype of the organism at a global level. This, in principle, arises from the complex exchanges of the metabolites among different compartments. Therefore, the understanding of metabolic processes of multicellular organisms at a global level requires the study of metabolic correlations among the tissue and biofluid metabolic profiles. Sophisticated chemometric techniques have been developed to address such problems. Montoliu et al. compared different unsupervised chemometric techniques in order to study intercompartmental metabolic analysis [18].

In this contribution, we report a NMR-based metabolite profiling followed by the multivariate statistical analysis strategy to delineate a distinct sexual dimorphism in the liver metabolic profile in the mouse model (BALB/c mouse infected with *Plasmodium berghei* ANKA) of malaria parasite infection. Furthermore, employing an unsupervised chemometric strategy (multiway principal component analysis- MPCA), we show the correlation of brain and liver metabolic profile with that of serum metabolic profile. The implications of these results are also discussed.

## 2. Materials and Methods

### 2.1. Animal Experiments

The animals in the study were treated in accordance with the prescribed guidelines of the institutional animal ethics committee.

### 2.2. Collection and Extract Preparation of the Sera, Brain and Liver Samples

The experiments were carried out in a similar fashion as described elsewhere [17]. Briefly, 24 inbred BALB/c mice (12 males and 12 females) aged 6 to 8 weeks were used for the study. Eight male and female mice were injected intraperitonally with 10^6^ *P. berghei *ANKA-infected erythrocytes. The remaining eight animals served as controls. Four infected males, four infected females and four control mice (two males and two females) were sacrificed on day 5 after infection. The rest of them were sacrificed on day 13 after infection. The animals were immediately dissected to extract the liver out. The liver samples were snap frozen and kept frozen until the preparation of perchloric acid extract as described earlier [17]. The sera and brain sample collection and extraction were done by the method described in [17].

### 2.3. NMR Spectroscopy of Tissue and Biofluid Extracts

^1^H NMR spectra were acquired on 700 MHz spectrometer (Bruker Biospin, Germany) equipped with broad band inverse probe using 2-dimethyl-2-silapentane-5-sulfonic acid (DSS) as an internal standard and the D_2_O as the field frequency lock at 300 K. The pulse sequence used included an excitation sculpting routine for the suppression of the water signal. 1024 transients were collected into 32,768 data points using a spectral width of 12.01 ppm resulting in an acquisition time of 1.94 s. A relaxation delay of 1 s was used between consecutive pulses. The FIDs so obtained were subjected to an exponential multiplication leading to an additional line broadening of 0.2 Hz. A sine bell apodization function was used followed by the Fourier transformation. The spectra were phase and baseline corrected manually and used for further data reduction.

2D COSY spectra were obtained for the identification of the metabolites in the following manner. In the direct dimension, 64 transients were collected with 256 increments in the indirect dimension. A QSINE function was used for processing with 2048 and 1024 data points in direct and indirect dimension, respectively.

### 2.4. Data Reduction

Two different data reduction procedures were followed. For the purpose of single tissue analysis (liver, for our case), the spectral region 9.5 ppm to 0.5 ppm was segmented in frequency regions of 0.04 ppm, and each bin was integrated using MestReC 4.7.0. The region corresponding to water and urea (4.5 ppm to 6.5 ppm) was excluded to avoid artifacts due to the water suppression and highly variable urea. Certain regions of the spectra are known to have high concentration metabolites with high variance that may mask the important features in the peaks with lower concentration. These regions were also excluded as described earlier [17]. The resulting integrals were normalized to total intensity and Pareto scaled to generate the working data matrix.

For the purpose of the intercompartmental correlation analysis, the binning procedure was done using Amix 5.0. Here, a binning width of 0.003 ppm was used, and only the water region was excluded (4.5 ppm to 5.1 ppm). The bins were integrated and normalized with respect to the total integral to generate the working data matrix.

### 2.5. Statistical Analyses

#### 2.5.1. Multivariate Analysis on Liver Samples

The liver spectra were subjected to the principal component analysis (PCA) and orthogonal partial least square-discriminant analysis (OPLS-DA), which were performed using Simca-P 12.0 (Umetrics, Sweden). Briefly, PCA is an unsupervised method which is used for revealing any inherent trend or pattern in the data set whereas, OPLS-DA is a supervised method. Here, the class entity towards the sample set is provided *a priori*, thereby showing the class-specific distinction. *Q* ^2^(cum) is the diagnostic parameter for the OPLS-DA model which represents the separation of the classes assigned. The models were set up in the following ways: (1) using data from uninfected males and day 5 post-infection males, (2) using data from uninfected males and day 13 post-infection males, (3) same as (1) for females, and (4) same as (3) for females. Initially, PCA was employed (data not shown) in order to find out any hidden trend in the data and to find outliers. This was followed by OPLS-DA. The visualization was aided by two-dimensional scores plot that shows the sample clustering in a two-dimensional space. The variables contributing towards the clustering in the scores plot were extracted using loadings S-plot and VIP plot. The metabolites were identified using Human Metabolome database (HMDB) along with the two-dimensional NMR spectral profiles.

#### 2.5.2. Peak Integration

After extracting the metabolites from the multivariate analytical techniques (PCA and OPLS-DA), individual peaks from the metabolites were selected in the one-dimensional NMR spectra, and they were integrated using Topspin 2.1. The crowding in the NMR spectral profile sometimes resulted in overlap of the peaks. In those cases, the metabolites are quoted together. The peak intensities of individual metabolites were calculated with respect to the intensity of internal standard DSS and normalized to the total tissue weight. Significance test for comparison were performed using Student's *t*-test. Early- and late-stage infection time points were compared with uninfected controls of the same sex. If the relative peak intensities of a metabolite were comparable (*P* > .15) between male and female uninfected samples, then a *t*-test was conducted to compare males and females at each post-infection time point. If the levels of the metabolite were different (*P* < .15) between uninfected males and females, then from each post-infection data point the value of the corresponding control average was subtracted. An average of these normalized values was taken and males and females were now compared for each post-infection time point to determine if the deviation from corresponding controls was significantly different between them.

#### 2.5.3. Intercompartmental Metabolic Correlation Analysis

Multiway PCA (MPCA) as described elsewhere [18] was used for the intercompartmental correlation analysis. Initially a Multivariate Curve Resolution-Alternating Least Square algorithm (MCR-ALS) was employed to check the relative contribution of the two relevant compartments. Broadly, two kinds of intercompartmental analysis were sought. (1) liver and serum and (2) brain and serum. In each of these categories, three different models were made for males and females, (a) controls, (b) day 5 after infection, and (c) day 13 after infection. This helps in the visualization of how the intercompartmental correlation changes as the disease progresses. For this purpose, a 3D matrix was prepared from the spectral integrals mentioned earlier using Matlab 7.0.1. In this matrix, the rows are the samples/animals, the columns are the spectral variables, and the third mode is the tissues/biofluids. This matrix was used for MPCA in Solo 5.8 (Eigenvector Inc.). The mathematical details of the method can be found elsewhere [18].

## 3. Results

Results for the serum and brain analysis have already been discussed elsewhere [17]. Here, we report the alteration of metabolic profile of liver and the intercompartmental correlation of brain and liver with sera.

### 3.1. Changes in Liver Metabolic Profile with Progression of the Disease

**Figure 1:**
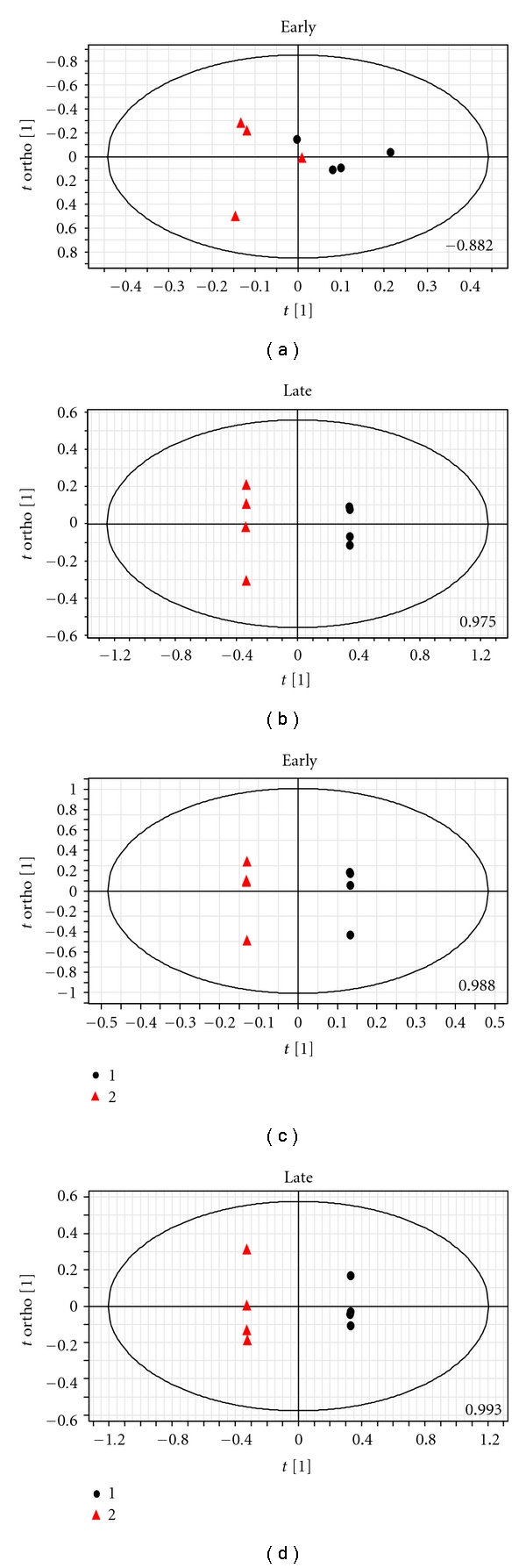
OPLS-DA scores plot based on the ^1^H NMR metabolic profile of the liver. The experiments consisted of 12 male and 12 female mice. Eight male and 8 female mice were injected with *P. berghei* ANKA. On day 5, 4 infected males, 4 infected females, 2 control males, and 2 control females were sacrificed, and the liver is extracted with perchloric acid (early-stage of infection). Again on day 13 (late-stage infection). A/B, early- and late-stage infection males are compared with male controls; C/D, early- and late-stage infection females are compared with female controls. In each plot, the *Q* ^2^(cum) is shown at the bottom. The red triangle—profile of the infected animals and the black dots—the control animals.

**Figure 2:**
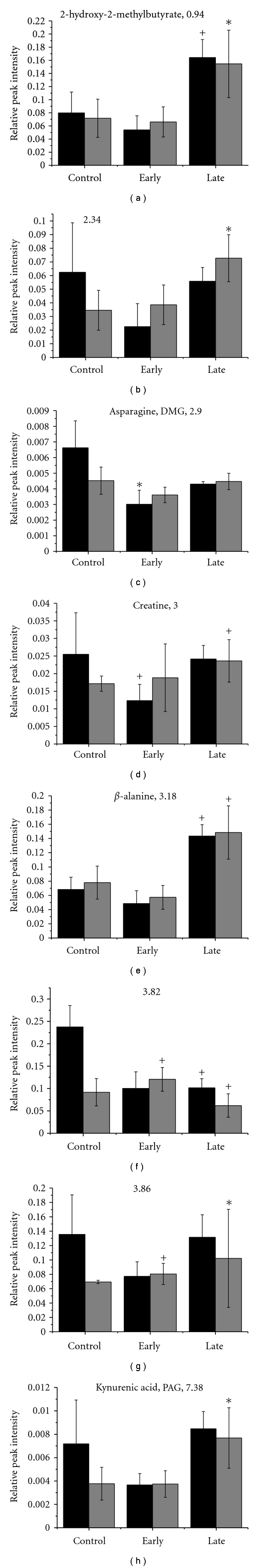
Perturbed metabolite levels in mouse liver at early- and late-stage malarial infection. All peak intensities have been calculated relative to the peak height of 0.132 mg/ml DSS present in every sample. Panels showing two metabolites represent overlapping ^1^H NMR resonances at the chemical shift mentioned. Where only chemical shifts are mentioned, identity of metabolites could not be confirmed. Black = males and grey = females. * indicates *P* < .05, ^+^ indicates *P* < .08 in comparison to the uninfected controls of the same sex.

**Figure 3:**
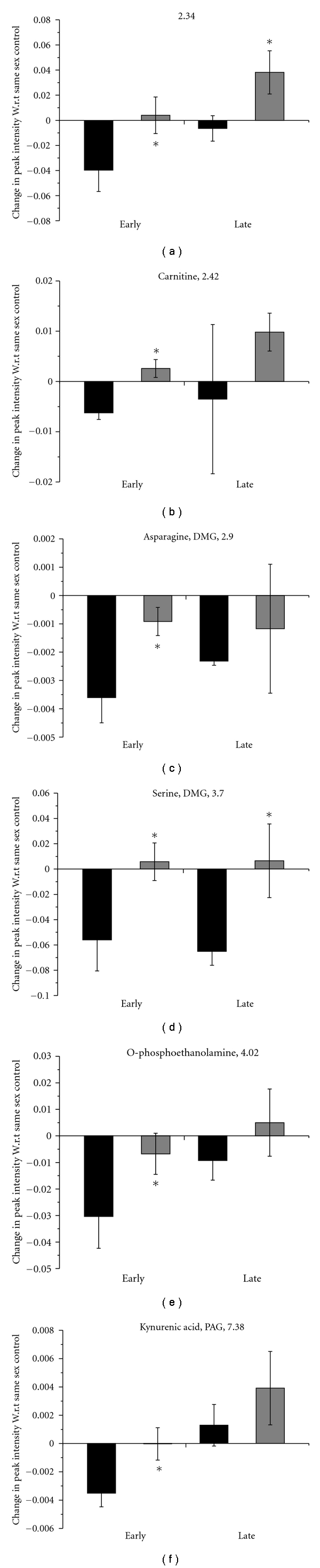
Perturbed metabolite levels in mouse liver at early- and late-stage malarial infection: comparison of effects in male and female animals. The average peak intensity for the metabolite in uninfected samples of the same sex was subtracted from individual peak intensities of infected animals at each stage of infection. The average change in intensity with respect to the same sex control is plotted here. * indicates *P* < .05, ^+^ indicates *P* < .08 in comparison to the males at each stage. Black = male, and grey = female. Panels showing two metabolites represent overlapping ^1^H NMR resonances at the chemical shift mentioned. Where only chemical shifts are mentioned, identity of metabolites could not be confirmed.

The liver extracts for the males showed no perturbation when the early infection time points were compared with uninfected animals (*Q* ^2^(cum) = −0.882). However, in the early infection time points, females showed a large change from that of uninfected controls (*Q* ^2^(cum) = 0.988). When the late-stage animals were compared with the uninfected controls, drastic changes were observed irrespective of the sex (*Q* ^2^(cum for males = 0.975 and *Q* ^2^(cum) for females = 0.993). The representative scores plot of the respective OPLS-DA models is shown in the Figure 1. The VIP plots (See Supplementary Information 1 available at doi:10.1155/2011/901854) and loadings S-plot (supplementary information 2) were further investigated in order to extract the most significant spectral variables/bins. These bins were further analyzed by the help of HMDB and two-dimensional NMR spectral profiles to generate the significant metabolites that contribute towards the pattern seen in the OPLS-DA models.  Table 1 shows a list of the metabolites perturbed in the liver as the disease progresses in both the sexes. However, as the early infection stage of the males showed insignificant variation (*Q* ^2^(cum)=−0.882, Figure 1(a)) from the control animals, hence, metabolites corresponding to that stage are not investigated. Further, the specific peaks from the one-dimensional NMR profiles were integrated with respect to the total intensity of the internal standard (DSS). Many of the metabolites identified from the OPLS-DA model showed statistically significant difference as the disease progresses (Figure 2). Moreover, analysis was done to compare some of the metabolites in the two post-infection stages which were different in the uninfected males and females to check whether they deviate significantly from the same sex control. These are shown in the Figure 3. OPLS-DA analysis of liver spectra showed some of the metabolites (Table 1) which were not observed in other tissues or biofluids [17]. However, although the OPLS-DA analysis showed a large difference in the early infection stage of females and uninfected controls (Table 1), some of the metabolites that were expected to be increased in the infected animals showed a large deviation in the VIP (supplementary information 1). Therefore, OPLS-DA was unable to predict the dramatic change in the early infected females with certainty; hence, the early-stage changes in the female liver remain to be characterized.

 In the late stage, the liver extracts showed an increase in 2-hydroxy-2-methylbutyrate and beta-alanine (Figures 2(a) and 2(e)) and a decrease in an unidentified compound at 3.82 ppm (Figure 2(f)) in both males and females. Along with these, females showed increase in kynurenic acid in the late stage (Figure 2(h)). Increase in two unidentified peaks at 2.34 and 3.86 ppm was also shown by the females (Figures 2(b) and 2(g)). 

In spite of the poor difference shown in the OPLS-DA between the male control and early-stage infected animals (Figure 1), some of the metabolites showed significant changes. These include asparagine, DMG, and creatine (Figures 2(c) and 2(d)), all of which are significantly decreased from the control animals in the early stage.

Comparisons of the individual metabolite level of the animals were also made with that of the controls (Figure 3), and several interesting results were obtained. This was done for the fact that several metabolites in the liver showed significant difference in their levels between male and female control animals (data not shown). Therefore, we investigated whether the post-infection alterations are different between males and females. Several of the changes observed in such analysis were often only in the early-stage. Females showed a slight increase from the corresponding controls, while males showed a decrease in the carnitine level at this stage (Figure 3(b)). Kynurenic acid levels in females did not change much from their controls whereas the males showed a decrease (Figure 3(f)). Asparagine, DMG and O-phosphoethanolamine levels were seen to be reduced significantly more in the early stage infected male liver samples as compared to females (Figures 3(c) and 3(e)). Some distinct sexual dimorphism in the alteration of the metabolite levels was observed. For example, an unidentified peak at 2.34 ppm showed decrease in the males while slight increase in the females compared to the corresponding controls (Figure 3(a)). Similar trend was observed for carnitine (Figure 3(b)), serine and DMG (Figure 3(d)).

Interesting here to note that is although the global metabolic profile of the male controls does not differ much from the male early infection animals, some of the individual metabolites show a significant alteration (Figures 2(c), 2(d), and 3). However, these alterations are possibly not enough to have an impact on the differential global metabolic profile (Figures 1(a) and 1(b)). 

### 3.2. Intercompartmental Correlation Analysis by Multiway PCA (MPCA)

MPCA has been established as useful tool for correlation analysis between tissues and circulating biofluids [18]. In this paper, we have used the technique to delineate the effect of disease progression on the correlation of metabolites across tissues/biofluids. We used earlier data of serum and brain [17] and the liver data described here to investigate the correlations of metabolites in brain and liver to that of serum with the disease progression in both the sexes. Earlier studies made by our group [17] showed that the brain metabolic profile is significantly altered in both the sexes. Although this model of malaria is not a cerebral variety, it is important to understand how the brain metabolic profile varies with the circulating biofluid. Initially, MCR-ALS algorithm (data not shown) was employed that showed appreciable contribution of one compartment on the spectrotype of the other. Earlier studies also found appreciable contribution of the blood serum spectrotype to the liver [18]. Further, MPCA models were built using the NMR metabolite profiles of the sera and the relevant tissue from a set of animals (e.g., 4 female controls, sera, and brain NMR metabolite profile). 12 such models were generated (control, early- and late-stage infection for males and females) for both types of correlations (brain-sera and liver-sera) to be addressed. The first two principal components (PCs) were calculated for each of them.  Table 2 shows the amount of variation explained by the models. All of them explained >90% of cumulative variance. The loadings in PC2 described the correlation of the NMR spectral peaks across the relevant tissue (either liver or brain) and serum.

**Figure 4:**
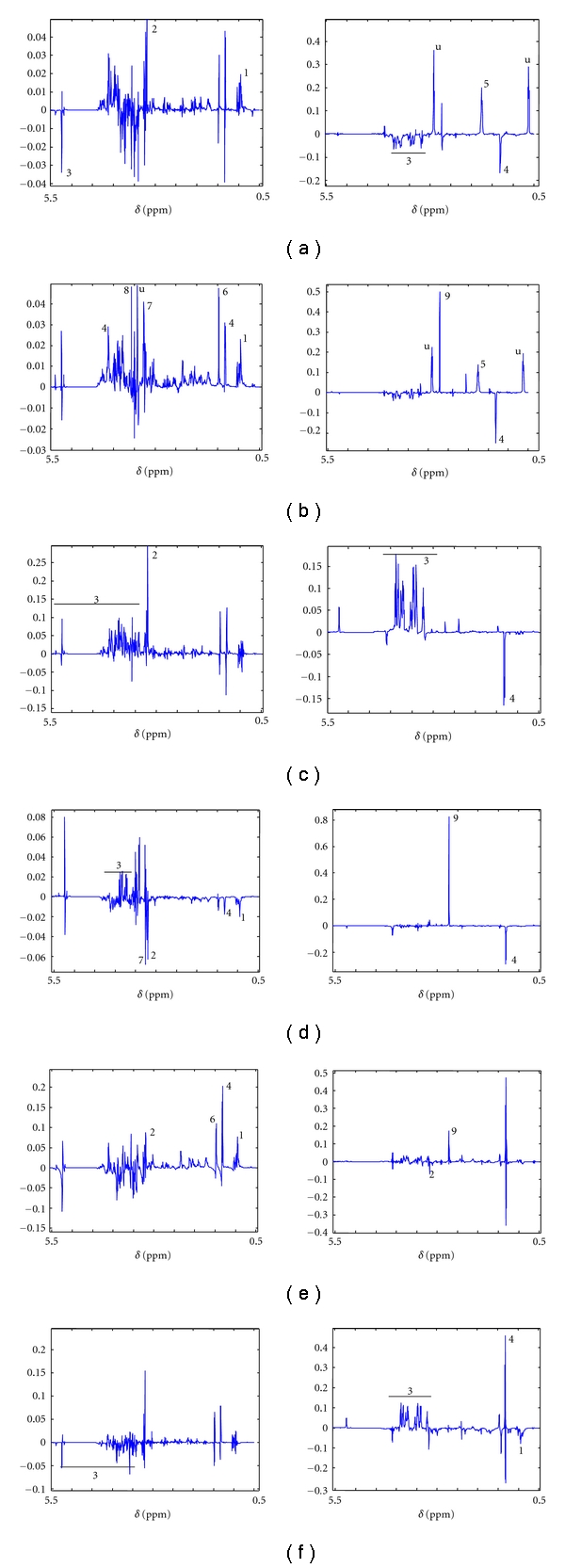
The liver and serum correlation profile depicted by MPCA PC2 loadings plot. The aliphatic region of the spectra is shown. (a–c) are females and (d–f) are the males. In each set, the left figure is the liver profile and the right one is the serum profile. The *y*-axis in each panel represents the loading axis. The compartmental loadings are obtained by cropping the model loading. (a/d): control, (b/e): early infection, and (c/f): late infection. Symbols: 1: branched chain amino acids, 2: choline, 3: glucose, 4: lactate, 5: lysine, 6: alanine, 7: trimethylamine oxide (TMAO), 8: glycine, 9: dimethylamine, and u: unidentified peak.

In female control animals, high concentration of branched chain amino acids (BCAA) (1) leucine, isoleucine, valine, choline (2) and a low concentration of glucose (3) in the liver were associated with low lactate (4), glucose (3) and high concentration of lysine (5) in serum (Figure 4(a)). There was a peak at 3.20 ppm (close to choline peak) which could not be identified. The females at early-stage infection showed essentially a similar serum profile to that of control animals, with an exception of a high dimethylamine (DMA) (9) in the serum (Figure 4(b)). However, the liver profile of these animals showed high branched chain amino acids (1), alanine (6), trimethylamine-N-oxide (TMAO) (7), glycine (8) and lactate (4) (Figure 4(b)). In the late-stage infection of the females, the liver showed a high choline (2) and glucose (3) which were associated with low lactate (4) and high glucose (3) level in the serum (Figure 4(c)).

Male control animals showed a different profile than that of the females. In the liver of the male control animals isoleucine (1 as BCAA), leucine (1 as BCAA), lactate (4), choline (2) and, TMAO (7) were low and glucose (3) was high. These were correlated to a low lactate (4) and high DMA (9) in the serum (Figure 4(d)). When the early-stage infection for the male animals was considered, the liver profile showed increase in BCAA (1), alanine (6), choline (2), and lactate (4) compared to the control animals, with the contribution of TMAO (7) becoming insignificant. The serum profile for this group of animals showed a high DMA (9) and low choline (2) level with respect to the liver profile (Figure 4(e)). The late-stage infection for the males was also relatively simple with low glucose (3) in liver being associated to high glucose (3), lactate (4) and low branched chain amino acids (1) in the serum (Figure 4(f)).

**Figure 5:**
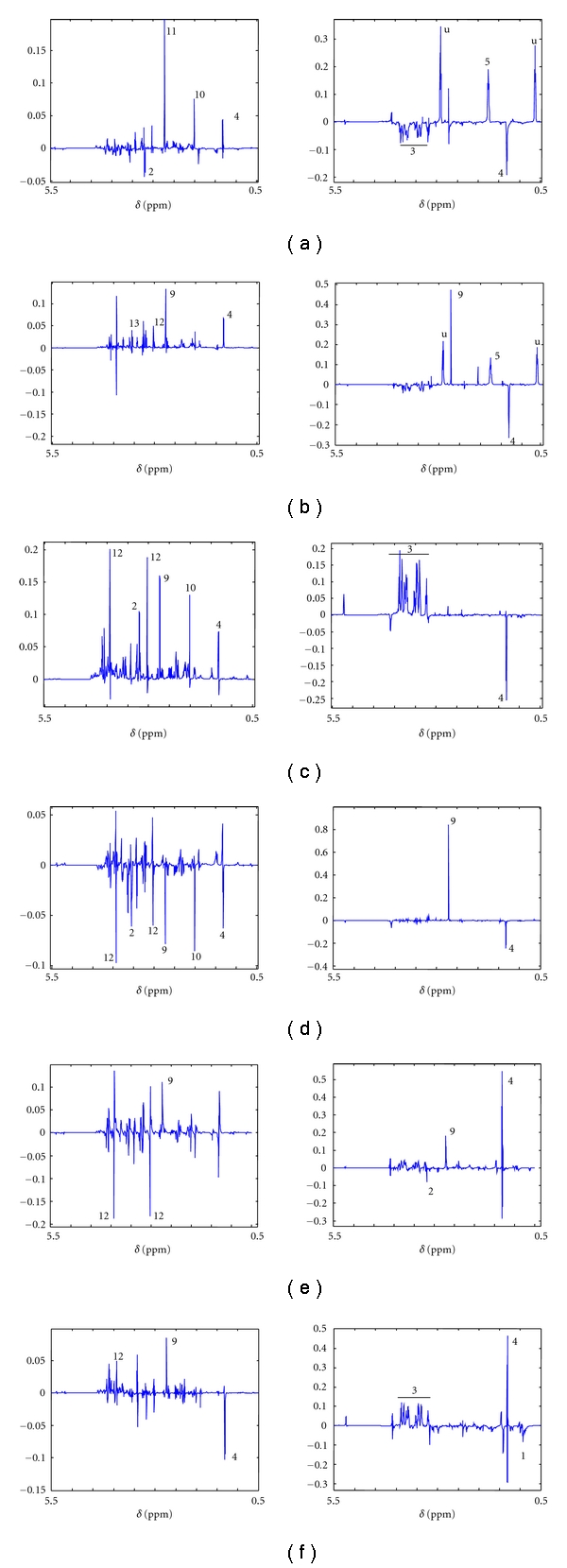
The brain and serum correlation profile depicted by MPCA PC2 loadings plot. The aliphatic region of the spectra is shown. The panels (a–c) are the females and (d–f) are the males. In each set, the left figure is the brain profile and the right is the serum profile. The *y*-axis in each panel represents the loading axis. The compartmental loadings are obtained by cropping the model loading. (a/d): control, (b/e): early infection, and (c/f): late-infection. Symbols: 10: N-acetylaspartate, 11: sarcosine, 12: creatine, 13: betaine, u: unidentified peak.

Our earlier studies (15) showed a significant deviation of the brain metabolic profile in the early-stage infection as well as the late-stage infection from the control animals. Thus, it would be interesting to see whether the serum- brain intercompartmental correlation is also affected with the disease progression. Therefore, the correlation of the brain metabolic profile with that of serum was assessed in order to delineate the crosstalk between these two biological compartments. The female control animals showed a high lactate (4), N-acetyl-aspartate (NAA, 10), sarcosine (11), and low choline (2) in the brain that correlated with high lysine (5) and low lactate (4) and glucose (3) in the serum (Figure 5(a)). In the early infection, the brain profile showed high lactate (4), DMA (9), creatine (12), and betaine (13). However, the serum profile remained similar to that of controls, although it showed a high DMA (9) (Figure 5(b)). In the late-stage, the brain metabolic profile showed high values of lactate (4), NAA (10), DMA (9), creatine (12), and choline (2). These were found to be significantly correlated with the high glucose (3) and low lactate (4) in the serum (Figure 5(c)).

The male metabolic intercompartmental analysis was also performed. The male controls, as expected, were different from that of females. They showed a low lactate (4), NAA (10), DMA (9), choline (2), and creatine (12) in the brain profile associated to a low lactate (4) and high DMA (9) in the serum (Figure 5(d)). The males showed change in the serum profile only in terms of a high lactate and low choline level, and the rest of the profile remained quite similar at the early-stage infection. At this stage, they showed a low creatine (12) and high DMA (9) in the brain (Figure 5(e)). The late infection stage showed a low lactate (4), high creatine (12), and DMA (9) in the brain and high lactate (4), glucose (3) and low BCAA in the serum (Figure 5(f)). 

Figure 4 (liver-serum correlation) and Figure 5 (brain-serum correlation) show a comprehensive visualization of all the increased and decreased metabolites in different compartments during the various stages of the disease. Important to note here is that the set of metabolites that were found to be significant from the OPLS-DA (Table 1, Figures 2 and 3 for liver metabolites and [17] for brain metabolites) study are different from those found from MPCA (Figures 4 and 5). It is important to realize at this point that OPLS-DA showed a set of metabolites that were significantly altered among the infected and control sets in a single tissue or biofluid. However, MPCA resulted in a set of metabolites which are significant in terms of the intercompartmental correlation.

## 4. Discussion

In this paper, we report our results on the alterations of the liver metabolism during the progress of malaria as well as the change in the correlation of the metabolic profile of the liver and brain with that of blood serum as the disease progresses. Murine model (BALB/c mice infected with *P. berghei *ANKA) using animals from both the sexes used for this purpose. Earlier results from our group [17] suggested that the host response towards the infection exhibits sexual dimorphism in the urine and serum metabolic profile. The alteration of the liver metabolism during the disease progression supports this fact. The liver profile in the female mice was altered in the early stage of the disease as compared to the males (Figure 1). Liver being the regulatory organ for metabolic activities, a drastic change in its metabolic profile in the early stage of the disease raises the possibility that the female hosts are able to respond to the infection by maintaining the homeostasis within them. This is supported by our earlier result that the female hosts show no early changes of the metabolic profile of the serum [17], while the males do. At the late stage, drastic changes in the metabolic alteration are observed irrespective of the sex of the host (Figure 1), which is anticipated because of the fact that the animals are very ill at this time point. 

Our earlier findings [17] indicated several alterations in the metabolic pathways during the progression of the disease. Among them, glucose metabolism, amino acid metabolism, kynurenine pathway, uracil degradation, and fatty acid metabolism were found to be significant. These observations were based on the alteration on the urine, sera, and brain metabolic profile. Our data on the liver metabolism reported here is in good agreement with that.

Glucose metabolism is known to be perturbed during malaria parasite infection largely due to the enhanced rate of glycolysis [19–21]. Lactic acidosis is an important pathophysiological feature of malaria. Our earlier results in the serum and brain metabolic profile showed a sexual dimorphism in lactate levels of those tissues. However, the comparison made with the liver metabolic profile does not show a pronounced increase in the lactate level. Instead, in the late-stage the lactate in the liver is shown to be decreased in males as well as females (Table 1). Enhancement of rate of glycolysis leads to an increased formation of pyruvate which may be transaminated to alanine. Thus, the change in the level of alanine is important in this regard. Alanine did not show the trend like lactate (Table 1). In the early-stage female animals, alanine is increased compared to the controls. The change in the alanine level is an indicator of the alteration of circulating amino acid levels. [17]. Liver being the metabolic regulatory organ, alteration in the level of the amino acids in the liver is expected. Along with alanine, the level of serine is also seen to be altered in the liver extracts. In the males, it is decreased, and in the females, the level is increased marginally when compared to the corresponding control thereby showing a sexual dimorphism (Figure 3(d)). Asparagine level also showed a significantly greater decrease in the males compared to the females with respect to the corresponding controls (Figure 3(c)). *Plasmodium sp. *are known to incorporate disproportionately a large amount of asparagine in their proteome in the ubiquitous low-complexity region [22]. Along with the amino acid mentioned, leucine levels was also increased in both males and females in the late stage (Table 1). However, alteration in the branched chain amino acids and aromatic amino acids were reported in context of *Trypanosome *infection also [23, 24].

Our earlier findings [17] showed a perturbation in the kynurenine pathway. We were the first to observe the increased excretion of kynurenic acid (KA) and quinolinic acid in the male infected mice. In the sera and brain, they showed a pronounced sexual dimorphism. When liver extracts are considered, KA exhibited a sexual dimorphism in the early-stage liver that its level in the infected male liver extracts decreased, while those in female did not (Figure 2(h)). The kynurenine pathway is activated by the immunological factor IFN-*γ;* therefore; it is systematically upregulated during the activation of the immune response under multiple disease condition [25]. Therefore, an interesting question that remains to be asked is whether the sexual dimorphism that we see in the pathway is a cause for the differential immunological activation between males and females.

Several components of fatty acid metabolic pathway are indicated to be perturbed in the liver extracts. A rise in fatty acid concentration in the male and female liver is indicated by the 0.9 ppm peak (Table 1). Oxidation of these fatty acids would entail generation of carnitine which we reported to be decreased in the urine of female mice [17]. This suggests a selectively higher retention of this metabolite for the cellular *β*-oxidation. The liver levels of carnitine also showed a sexual dimorphism in the early stage (Figure 3(b)). Whereas the males showed a slight decrease, the females demonstrated a slight increase from the corresponding controls. Along with this, choline was indicated to be increased in the early-stage infected females (Table 1). O-phosphoethanolamine was detected in the liver sample to show a sexual dimorphism in the late stage (Figure 3(e)) along with 2-hydroxy-2-methylbutyrate which was increased significantly in both the sexes in the late stage (Figure 2(a)).

The crosstalk (correlation) of metabolites across organs and biofluids is an important parameter in their changing dynamical interaction during disease progression. For this purpose, we analyzed the correlation of the liver and brain metabolic profile with that of blood serum. Previously, we delineated the effect of disease progression on the brain metabolic profile individually [17]. Important to note is that at the late stage of infection, the correlation profiles look drastically different than that of the controls (Figures 4(c), 4(f), 5(c), and 5(f)). This is expected because of the enhanced severity of the disease. Therefore, we would concentrate more on the early-stage correlation profile. As noted earlier the liver serum correlation of the female animal is distinct from that of the males in the control animals (Figures 4(a) and 4(d)). Initial study on the liver profile of the control animals showed no distinction between the two sexes (data not shown), and similar results were observed for the serum profile [17]. Thus, it is evident that the dynamic intercompartmental correlation is maintained differently in the two sexes which result in similar global metabolic profile of the individual compartment. For example, in both of them, the amino acids (1) in the liver are anticorrelated to the glucose (3) in the liver. This possibly refers to the gluconeogenetic pathway. However, the females and males show different modes of correlation. Males showed a low amino acid concentration to be correlated to a high glucose concentration (Figure 4(d)) whereas females showed the opposite (Figure 4(a)). In addition, males showed a low serum lactate (4) to be correlated with high liver glucose (3) (Figure 4(d)), and females showed a low serum lactate (4) to be correlated with low liver glucose (3). This suggests a complex glycolytic-gluconeogenetic relationship which keeps the global metabolic profile of the individual tissue and/or biofluid more or less similar between the two sexes. 

The data presented here also suggests that this correlation is compromised during the course of the disease in some way or another. For the females in the early stage, the serum compartment of the correlation profile looks more or less the same except for an increased contribution from the DMA (9), whereas there are notable changes in the liver segments of the profile (Figures 4(a) and 4(b)). However, in the males, both serum and liver segment showed notable changes (Figures 4(d) and 4(e)). Therefore, we observe a sexual dimorphism even in the intercompartmental correlation status when the liver and serum are considered. This may implicate that the amount of circulating metabolites in the females remain under homeostasis, while their metabolism in the liver is altered drastically. This supports the hypothesis that there is significant amount of change in the early-stage female liver in order to maintain the homeostasis in the circulating fluid in response to the pathogen. In the female early-stage infection an increased alanine (6), TMAO (7), glycine (8), and lactate (4) in the liver were correlated with an increased DMA (9) in the serum. DMA is a metabolic product of TMAO, so an increased DMA in the serum can be correlated with an increased TMAO in the liver. TMAO has been reported to be associated with kidney disease [26] and is used by the body as an osmolyte to counter-act the accumulation of urea due to renal failure. This refers to the fact that the females can or at least try to maintain other organs under functional state at the early stage of the disease. As noted earlier, lactate did not show a significant contribution towards the differentiation of infected and control animals; however, in the correlation analysis, we observe an inverse relation of the liver and serum lactate for the early-stage females (Figure 4(b)). Here, a relative buildup in the liver lactate is associated with the depletion of the serum lactate. Lactate is a gluconeogenic precursor in the liver. It is possible that the heavy utilization of glucose in the blood is counteracted by the host by enhancing the rate of gluconeogenesis in the liver. However, if that is the case, then one would expect an opposite relation between the liver and serum lactate to that we see here. Gluconeogenesis is enhanced in the severe malaria patients [27]. This study also reported an enhancement of serum lactate. Our result, therefore, suggests a more intricate functional and spatiotemporal relationship between the liver and serum lactate. For example, it might be possible that an alteration in the Cori cycle pathway changes the uptake of lactate from serum into liver. However, the details of this process remain to be investigated. In males, however, both the liver and serum profile changes in the early stage of infection (Figure 4(e)), referring to the fact that the males are unable to maintain the blood homeostasis. Moreover, the liver metabolism of these animals is altered drastically. Here, the choline of liver is found to be anticorrelated to the choline of serum. Precisely, an increased choline in the liver is associated with a decreased choline in the serum. This may be due to the fact that the intraerythrocytic parasite causes a 5-fold increase in the phospholipid content of the parasitized cell [28], which may eventually lead to a phospholipid degradation in the liver of the host, thereby generating more of the phosphocholine components.

As far as the brain to serum correlation analysis is concerned, for the females, the contribution from the serum towards the brain-serum crosstalk does not alter much; however, the brain profile did change notably (Figures 5(a) and 5(b)). For the males, both the brain and serum profile was altered (Figures 5(d) and 5(e)), although the early-stage brain profile looks similar to that of control animals. Earlier report showed that the brain profile in the early-stage males and females did not change significantly [17]. However, this data suggests that the brain profile is maintained by the aid of a complex relationship with the serum. In the females, as noted earlier, the homeostasis of the serum is maintained, while for the males, the brain profile is maintained at the cost of serum homeostasis. In females, the brain showed a relative increase in the lactate and creatine, whereas the males showed a relative decrease of creatine. This indicates that the energy metabolism of the brain is altered in the early stage of the infection in both sexes. However, the alteration in the energy metabolism probably bears different meanings in the two sexes. In the males, this is an effect of the maintenance of brain homeostasis at the cost of serum homeostasis, whereas, for the females it is the other way around. 

This paper presents the initial understanding of the complex interplay of the various biological compartments during the progression of malaria in mice. This, therefore, opens up the possibility of a greater insight into the changes in the metabolic pathways as a response to malarial parasite infection.

## Figures and Tables

**Table 1:** Metabolites perturbed in the mouse liver during infection with *P. berghei* ANKA. Italicized metabolites could not be confirmed. Cystine, serine and phosphoserine could not be confirmed because their crosspeaks occur in very crowded regions of the spectrum. Presence of fatty acids cannot be confirmed due to low concentrations.

Increased compound	Chemical shift	VIP value	Loading	Decreased compound	Chemical shift	VIP value	Loading
Females early stage							
N-methyl-a-aminobutyric acid, Ribonolactone	3.82	3.01	0.242	Beta-alanine	3.18	5.66	−0.455
Dimethylglycine	3.7	2.85	0.229	O-phosphoethanolamine	4.02	2.5	−0.201
Phosphocreatine, Creatine, Betaine	3.9	2.72	0.219	Unidentified	3.66	2.12	−0.171
Unidentified	3.46	2.58	0.207	Sarcosine	3.62	2.84	−0.228
Alanine, methylacetoacetic acid, acetylcholine	3.74	1.91		Choline, Creatinine	4.06	2.37	−0.191
Choline,	3.5	1.5	0.153				
3-hydroxyisovaleric acid, 4-pyridoxic acid	2.34	1.62	0.130				

Female late stage							
2-hydroxy-2-methylbutyric acid	0.94	3.01	0.242	Dimethylglycine	3.7	3.34	−0.268
2-ethylacrylic acid	0.98	2.41	0.194	N-methyl-a-aminobutyric acid, ribonolactone	3.82	3.34	−0.268
Diethyl-thiophosphate, O-phosphoethanolamine, Phosphoserine	3.98	2.38	0.191	Unconfirmed	3.46	3.28	−0.263
Beta-alanine	3.18	2.21	0.178	N-methyl-a-aminobutyric acid,3-mercaptopyruvic acid	3.86	3.05	−0.245
Dimethyl-sulphide, Acetyl-phosphate, Amino-acetone, cis-2-methylaconitate	2.10	2.15	0.173	Alanine, methylacetoacetic acid, acetylcholine	3.74	2.73	−0.219
Putrescine, L-leucine, 2-hydroxy-2-methylbutyric acid	1.7	2.03	0.163	Lactate,long chain fatty acids	1.3	2.18	−0.175
3-hydroxyisovaleric acid, 4-pyridoxic acid	2.34	1.87	0.150	Glycine	3.54	2.13	−0.171
2-ethylacrylic acid, stearic acid	1.02	1.82	0.146	lactic acid, N-acetyl-L-alanine	4.1	1.74	−0.140

Males late stage							
Beta-alanine, Cystine	3.18	3.26	0.262	N-methyl-a-aminobutyric acid,Ribonolactone	3.82	3.52	−.282
2-hydroxy-2-methyl-butyric acid	0.94	3.00	0.241	N-methyl-a-aminobutyric acid,3-mercaptopyruvic acid	3.86	3.38	−.271
2-ethylacrylic acid	0.98	2.44	0.196	Serine, Dimethylglycine	3.7	3.13	−.251
Dimethyl-sulphide, Acetyl-phosphate, Amino-acetone, cis-2-methylaconitate	2.1	1.98	0.159	Alanine, methylacetoacetic acid, acetylcholine	3.74	2.99	−.20
Putrescine, 2-hydroxy-2-methylbutyric acid, L-leucine	1.7	1.95	0.156	Phosphocreatine, Creatine, Betaine	3.9	2.44	−.196
2-ethylacrylic acid, stearic acid	1.02	1.77	0.142	cis-aconitic acid	3.46	2.33	−.187
Long-chain fatty acids	0.9	1.73	0.139	lactic acid, N-acetyl-L-alanine	4.1	1.98	−.160
O-phosphoethanolamine, phosphoserine	3.98	1.73	0.139	Alanine, Acetylcholine, guanidinoacetic acid	3.78	1.71	−.136

**Table 2:** MPCA model statistics in terms of total explained variances by two PCs calculated in percentage.

Model	Total variation explained (%)
Liver-serum correlation, female	
Control	93.17
Early infection	98.15
Late infection	99.25

Liver- serum correlation, male	
Control	96.40
Early infection	99.02
Late infection	98.08

Brain- serum correlation, female	
Control	94.55
Early infection	98.81
Late infection	99.25

Brain- serum correlation, male	
Control	96.72
Early infection	98.02
Late infection	98.58
